# Salivary androgens in adolescence and their value as a marker of puberty: results from the SCAMP cohort

**DOI:** 10.1530/EC-23-0084

**Published:** 2023-11-08

**Authors:** Supitcha Patjamontri, Alexander Spiers, Rachel B Smith, Chen Shen, Jo Adaway, Brian G Keevil, Mireille B Toledano, S Faisal Ahmed

**Affiliations:** 1 Developmental Endocrinology Research Group, University of Glasgow, Royal Hospital for Children, Glasgow, UK; 2 Division of Endocrinology and Metabolism, Department of Pediatrics, Faculty of Medicine Siriraj Hospital, Mahidol University, Bangkok, Thailand; 3 MRC Centre for Environment and Health, Imperial College London, London, UK; 4 NIHR Health Protection Research Unit on Chemical Radiation Threats and Hazards, Imperial College London, London, UK; 5 National Institute for Health Research (NIHR) Health Protection Research Unit in Environmental Exposures and Health, Imperial College London, London, UK; 6 Mohn Centre for Children’s Health and Wellbeing, Imperial College London, London, UK; 7 Department of Clinical Biochemistry, Manchester University NHS Foundation Trust, Manchester Academic Health Science Centre, The University of Manchester, Manchester, UK

**Keywords:** androstenedione, liquid chromatography-mass spectrometry (LC-MSMS), pubertal development scale (PDS), saliva, testosterone

## Abstract

**Context:**

Salivary androgens represent non-invasive biomarkers of puberty that may have utility in clinical and population studies.

**Objective:**

To understand normal age-related variation in salivary sex steroids and demonstrate their correlation to pubertal development in young adolescents.

**Design, setting and participants:**

School-based cohort study of 1495 adolescents at two time points for collecting saliva samples approximately 2 years apart.

**Outcome measures:**

The saliva samples were analyzed for five androgens (testosterone, androstenedione (A4), 17-hydroxyprogesterone, 11-ketotestosterone and 11β-hydroxyandrostenedione) using liquid chromatography-mass spectrometry; in addition, salivary dehydroepiandrosterone (DHEA) and oestradiol (OE2) were analysed by ELISA. The pubertal staging was self-reported using the Pubertal Development Scale (PDS).

**Results:**

In 1236 saliva samples from 903 boys aged between 11 and 16 years, salivary androgens except DHEA exhibited an increasing trend with an advancing age (ANOVA, *P* < 0.001), with salivary testosterone and A4 concentration showing the strongest correlation (*r* = 0.55, *P* < 0.001 and *r* = 0.48, *P* < 0.001, respectively). In a subgroup analysis of 155 and 63 saliva samples in boys and girls, respectively, morning salivary testosterone concentrations showed the highest correlation with composite PDS scores and voice-breaking category from PDS self-report in boys (*r* = 0.75, *r* = 0.67, respectively). In girls, salivary DHEA and OE2 had negligible correlations with age or composite PDS scores.

**Conclusion:**

In boys aged 11–16 years, an increase in salivary testosterone and A4 is associated with self-reported pubertal progress and represents valid non-invasive biomarkers of puberty in boys.

## Introduction

Pubertal progress is accompanied by a steady increase in a wide range of sex steroids that are produced by the adrenal glands and the gonads. The increase of serum testosterone and free testosterone during puberty is well-known and there are several studies correlating testosterone to Tanner stages of puberty (1, 2). Most circulating sex steroids are bound to proteins with only 0.5–3.0% available in plasma as an unbound steroid and it is this free form which exerts steroid activity at the level of the target tissue (3). Free sex steroids including androgens are small lipophilic compounds and can also enter the salivary glands from the capillaries by passive diffusion and there is a good correlation between salivary and serum androgen concentrations from previous studies (4, 5, 6).

Salivary testosterone has been described as a marker of diagnosis or therapeutic control in male hypogonadism (7, 8). In addition, salivary testosterone, androstenedione (A4), 17-hydroxyprogesterone (17-OHP), 11-ketotestosterone (11-KT), 11β-hydroxyandrostenedione (11-OHA4) showed a strong correlation with their serum levels in 21-hydroxylase deficiency congenital adrenal hyperplasia (CAH); therefore, measuring these salivary androgens could be non-invasive markers for treatment monitoring in CAH patients (4, 9). However, these situations have rarely been correlated to pubertal status. In non-clinical health research, salivary androgens, particularly testosterone, have been generally measured by radioimmunoassay (RIA) (10, 11, 12, 13, 14). However, RIAs are not well suited for the measurement of salivary androgens as, in addition to the need for a high level of sensitivity, the presence of other steroid hormones as well as precursors and metabolites in the assay matrix leads to a greater chance of cross-reactivity. Liquid chromatography-mass spectrometry (LC-MS/MS) offers higher sensitivity and specificity with greater accuracy for steroid hormone measurement as well as a lower limit of quantification (15, 16) and has been used to describe age-related changes in salivary testosterone in children, adolescents and adults (15, 16, 17, 18, 19). However, there is scarce information on the wider range of salivary sex steroids including those that are involved in the alternative pathways of steroid synthesis (20), and there is even less information on their relationship to puberty. Other considerations such as the effect of long-term storage of samples and the timing of sample collection have also not been studied sufficiently. The primary objective of the current study was to investigate age-related variations in a wide range of salivary steroids in healthy school children and adolescents and explore their relationship to pubertal development, with secondary objectives of investigating the effect of sample collection time and the stability of the long-term storage of the samples for measuring the sex steroids in saliva.

## Methods

### Study setting and population

**Figure 1:**
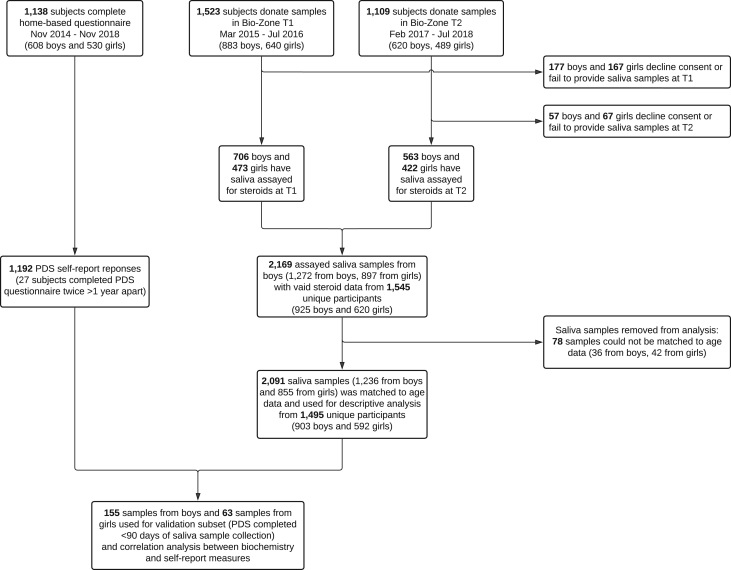
Structure of SCAMP cohort data relevant to this study. ‘Bio-Zone’ describes school collections where non-invasive biological samples (urine and saliva) and anthropometric measurements were collected. T1 and T2 relate to periods spanning the first wave (between March 2015 and July 2016) and the second wave (between February 2017 and July 2018) of Bio-Zone data collection.

The current study was part of a large school-based adolescent cohort study, SCAMP, conducted in 39 secondary schools across Greater London, UK (21). In addition to completing a series of questionnaires that included demographic details and concomitant medications, this sub-study of participants at 12 schools also took part in SCAMP ‘Bio-Zone’ sessions which involved providing saliva and urine samples and collection of anthropometric measurements. For the analysis of salivary steroids in the present study, a total of 925 boys and 620 girls had at least one saliva sample analysed at one of two time points, T1 (between March 2015 and July 2016) and T2 (between February 2017 and July 2018). Figure 1 shows the cohort data relevant to this study. There were no reports of steroid hormonal therapy or the use of contraceptives. Saliva samples were collected from participants during school hours between March 2015 and July 2016 (T1); 706 boys with a median age of 12.3 years (range, 11.3, 13.2) and 473 girls with a median age 12.3 years (11.1, 13.2). Approximately 2 years later (T2) between February 2017 and July 2018, 563 boys with a median age of 14.3 years (13.4, 15.8) and 422 girls with a median age of 14.3 years (13.4, 15.7) from 10 of the 12 schools had further saliva samples collected when participants were aged 13-15 years.

### Self-reported pubertal development

Most SCAMP questionnaire data collection was conducted in schools, but for reasons of school time limitations and question sensitivity, some additional questions were asked via a home-based online questionnaire. All SCAMP participants were invited to complete an online questionnaire at home, which included 14 questions on puberty (7 for each sex) based on the validated Pubertal Developmental Scale (PDS) (22). Previous studies demonstrated that the association between professional-rated Tanner stage and PDS self-report was moderate to high and that PDS self-report can be used for pubertal assessment in research without the need for Tanner evaluation when the precise concordance is not required (23, 24, 25, 26). Of the total SCAMP cohort, 608 boys and 530 girls (1138 in total) reported their pubertal development using the PDS. Participants had the option of repeating the questionnaire at a later date; if two questionnaires were completed within 12 months by the same participant, only the first completed was used for analysis (Fig. 1). The questionnaire asked participants to self-report secondary sexual characteristic development from 1 (development not started) to 4 (development completed). Scale items included growth in height, pubic hair, axillary hair and skin changes for both genders. Boys were asked additional items on deepening of voice and facial hair and girls were asked items on breast development and onset of menarche. PDS scores were then converted using an algorithm developed by Crockett (1988, unpublished) to one of five PDS-derived pubertal categories to summarize participants’ pubertal development: pre-pubertal, early pubertal, mid-pubertal, late-pubertal and post-pubertal. To evaluate correlations between salivary sex steroid concentration and self-reported development, we selected questionnaires completed less than 90 days of a saliva collection, giving a validation sample of 155 and 63 questionnaire/sample pairs from boys and girls, respectively.

### Sample collection, stability analysis and assays

Saliva samples were collected during school hours directly into sterile polypropylene vials and the time of collection was recorded. To prevent contamination and dilution of the saliva sample, participants were not permitted to eat or drink 30 min before saliva sampling began. Saliva was collected by passive drool into 7 mL polypropylene containers with polyethylene caps; participants were instructed to drool at least 2 mL. No salivary stimulants were used to provoke salivary flow. Samples were then transported by Thermoporter before being aliquoted and stored long term at −20°C until cryo-shipment to the laboratory for analysis. To investigate long-term stability of the analytes, saliva was also collected from 35 healthy adult volunteers (18 female), aliquotted, cryo-shipped to the laboratories and stored at −20°C until biochemical analysis. One aliquot from each of the samples was analysed, and a further aliquot from each sample was analysed at 4 further time points up to a maximum of 608 days for steroids assayed by LC-MS/MS and 664 days for steroids assayed by ELISA immunoassay (Salimetrics Assay, State College, PA, USA).

Saliva samples for males were analysed for testosterone, A4, 11KT, 17-OHP, 11OHA4 and DHEA. Saliva samples for females were analysed for OE2 and DHEA only. Sample assay methods were the same for samples collected from both SCAMP participants and healthy adult volunteers, as follows. Testosterone, A4, 17-OHP, 11-OHA4 and 11-KT concentrations were determined by LC-MS/MS. The lower limit of quantification was 5 pmol/L for testosterone; 10 pmol/L for A4; 12.5 pmol/L for 17-OHP; 45 pmol/L for 11-OHA4 and 6 pmol/L for 11-KT. The inter- and intra-assay coefficient of variation (CV) at the lower limit of quantification was 13.6% (between-batch) and 7.5% (within-batch CV) for testosterone; 12.8 and 3.3% for A4; 9.4 and 3.6% for 17-OHP; 6.3 and 3.4% for 11-OHA4 and 11.0 and 6.9% for 11-KT. OE2 and DHEA concentrations were determined by ELISA (Salimetrics Assay). The lower limit of quantification was 0.365 pmol/L for OE2 and 0.0174 nmol/L for DHEA. The inter-assay CV was 8.2 and 12.5%, and the intra-assay CV was 7.1 and 5.6% for OE2 and DHEA, respectively. To reduce bias when calculating reference ranges or conducting statistical tests, concentrations measured below the lower limit of quantification were imputed as the lower limit of quantification multiplied a factor of 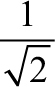 (27).

### Statistical analysis

All salivary samples were included in the analysis, i.e. no samples were removed from the analysis due to being outliers. Reference ranges for all the measured steroids stratified by sex were calculated for 11–16 years by year increment. One-way ANOVA and post-hoc Games-Howell test were conducted to assess for differences in mean scores by age. Pearson correlation was used to investigate the correlation of steroid concentration with age. Nonparametric quantile regression was used to fit percentile curves at 2.5, 16, 50, 84 and 97.5%, conditional on age for each sex steroid separately (28). Correlations were evaluated between salivary sex steroid concentration and pubertal development from self-reported PDS scores and PDS-derived pubertal category ranks in participants who provided samples and completed self-report questionnaires. Spearman rank correlations were used to analyse the relationship between each PDS domain score, composite PDS (mean of all PDS domain scores), PDS-derived pubertal category ranks and salivary steroid concentrations. Salivary androgens exhibit diurnal variation and the times reported for specimen collection from previous studies are usually between 07:00 and 11:00 h (4, 6, 13, 15, 16, 19). In the present study, to assess if diurnal changes affected the inferences, the same correlation analysis (between salivary steroid concentration and PDS) was conducted with subgroups of samples that were collected in early morning or later in the school day; cut-off of 11:00 h was used based on previous studies (19). To further assess diurnal change, Wilcoxon–Mann–Whitney *U* test was conducted in boys (stratified by age in 1-year intervals) to compare differences in concentrations for testosterone and A4 with respect to time of collection, again before and after 11:00 h. The capacity of each biomarker to predict marked voice change (PDS score 3 and above) was evaluated using receiver operator characteristic (ROC) curve analysis, which provided sensitivity and specificity, area under the curve (AUC) measures and optimum cut-off values from bootstrapped maximized Youden index. For stability analysis, linear mixed effects models with random intercepts for subjects were fitted to test for the significance of the effect of storage time. For biomarkers that showed a significant effect of storage time, post-hoc estimations of degradation or accumulation kinetics were conducted. Prior to model fitting, the data were normalized to a percentage of the baseline (time = 0) concentration. For steroids that showed significant degradation, we assumed their kinetics followed a single first-order (SFO) exponential decline, i.e. that the rate of degradation was proportional to the concentration, a common kinetic profile of biomarkers in storage (29). For steroids that showed a significant increase in concentration, the data were fitted to linear models. Bayesian mixed effects models were fitted using MCMC estimation with Stan (30) using the brms package (31) to model between-subject heterogeneity of rate slopes. For this study, the posterior mean annual change in concentration is reported for each steroid deemed unstable. For degradation SFO models estimates of the population-average SFO rate constant (k), the half-life or time required for steroid to halve in concentration (t_1/2_) and their respective 95% credible intervals for each unstable biomarker were also evaluated. All analyses were conducted using IBM SPSS Statistics (Version 20) predictive analytics software and R version 4.0.3, with packages *brms*, *cutpointr* and *np* (31, 32, 33).

### Ethics

The North West Haydock Research Ethics Committee approved the SCAMP study protocol and subsequent amendments (ref 14/NW/0347). School headteachers consented to participation in SCAMP. Parents and adolescents were provided in advance with written information about the study and were given the opportunity to opt out of the research at any time. The study was conducted in accordance with the Declaration of Helsinki. Saliva samples from healthy adult volunteers that were used for the stability analysis were collected under the framework of the Imperial College Healthcare Tissue Bank which is approved by Wales REC3 to release human material for research (17/WA/0161). Consent has been obtained from each healthy adult participant of the stability analysis after a full explanation of the purpose and nature of all procedures used. The samples for this project were issued from sub-collection reference number (MED_MT_19_036).

## Results

### Age- and sex-related data

**Figure 2:**
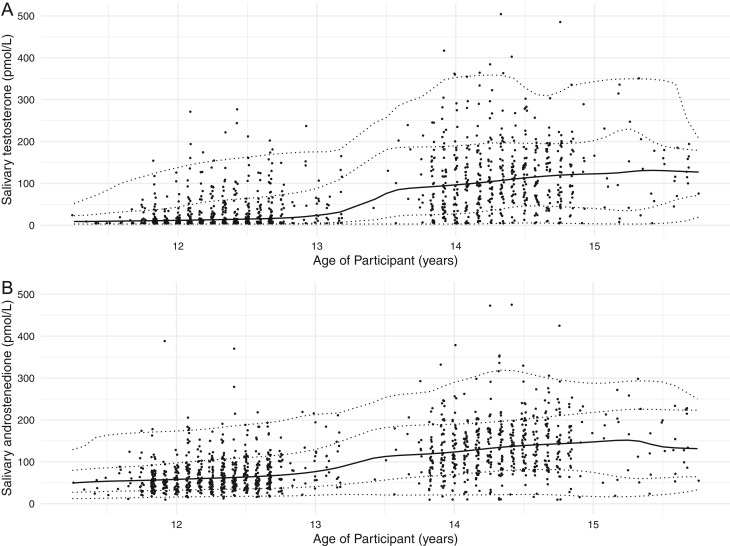
Scatter plot demonstrating the distribution of (A) salivary testosterone and (B) androstenedione concentrations in boys. The solid lines represent the median, and the dash lines represent the estimated ±1 and ±2 z-scores.

A total of 1236 saliva samples were available from 903 boys aged between 11 and 16 with a median age of 12.3 years (11.3, 13.2) and 14.3 years (13.4, 15.8) at T1 and T2, respectively. A total of 333 boys gave saliva samples at both T1 and T2. In these boys, all salivary androgens (testosterone, A4, 17-OHP, 11-KT, 11-OHA4) except DHEA exhibited an increasing trend with advancing age (ANOVA, *P* < 0.001). Salivary testosterone concentrations revealed the highest correlation with age (*r* = 0.55, *P* < 0.001, *n* = 1166) followed by A4 (*r* = 0.48, *P* < 0.001, *n* = 1167), 11-KT (*r* = 0.30, *P* < 0.001, *n* = 1145), 17-OHP (*r* = 0.27, *P* < 0.001, *n* = 1139), 11-OHA4 (*r* = 0.15, *P* < 0.001, *n* = 1161). Salivary DHEA concentration did not demonstrate a significant trend with advancing age in boys or girls (ANOVA, *P* = 0.52 and 0.29, respectively). Eight hundred and fifty-five samples were available from 592 girls between 11 and 16 with a median age of 12.3 years (11.1, 13.2) and 14.3 years (13.4, 15.8) at T1 and T2, respectively. A total of 262 participants gave saliva samples at both T1 and T2. For girls, salivary OE2 and DHEA concentrations showed a negligible correlation with age (*r* = 0.12, *P* = 0.001 and *r* = 0.05, *P* = 0.167, respectively). Table 1 and Fig. 2 show percentiles for salivary testosterone and A4 concentrations in boys aged between 11 and 16 years. Supplementary Table 1, 2 and 3 (see section on supplementary materials given at the end of this article) show percentiles for all other salivary androgens in boys and Supplementary Table 4 show percentiles for salivary DHEA and OE2 in girls aged 11–16 years.

**Table 1:** Percentile for salivary testosterone and androstenedione concentrations in boys aged 11–16 years.

Age (years)	*n*	Percentile				
		2.5	16	50	84	97.5
**Salivary testosterone (pmol/L)**						
11–12	125	3.5	3.5	6.0	28.5	75.3
12–13	479	3.5	3.5	11.0	62.5	161.0
13–14	119	4.9	16.7	66.0	166.8	255.4
14–15	407	3.5	35.0	107.0	195.0	352.1
15–16	37	7.6	43.8	128.0	196.9	337.5
11–16	1167	3.5	3.5	41.0	148.0	278.8
**Salivary androstenedione (pmol/L)**						
11–12	125	20.1	29.0	51.0	78.2	159.1
12–13	479	21.0	36.0	62.0	108.0	184.0
13–14	119	31.5	56.0	110.0	185.2	245.1
14–15	407	24.0	78.9	138.0	198.0	297.1
15–16	37	23.5	55.8	154.0	225.2	274.6
11–16	1167	21.0	40.0	87.0	167.0	251.7

### Specimen collection time and salivary hormone concentrations

**Figure 3:**
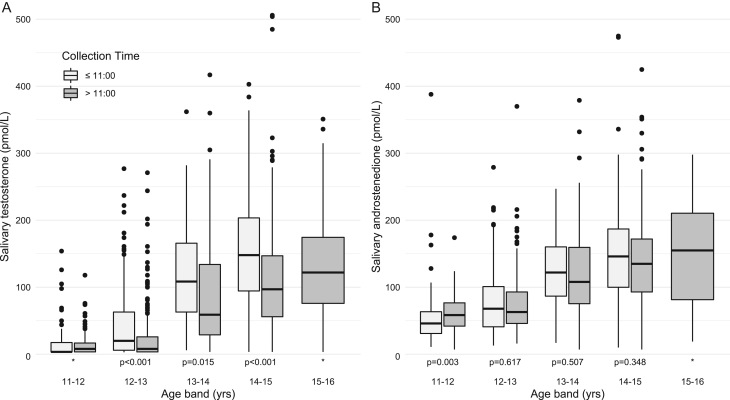
Boxplots showing salivary concentrations of (A) testosterone and (B) androstenedione collected from boys before and after 11:00 h, stratified by age. *P*-values for Wilcoxon–Mann–Whitney *U* test comparing each steroid distribution between time periods. *Wilcoxon–Mann–Whitney *U* test not performed. For testosterone from within the 11–12 age band, this was because the majority of samples were left-censored, i.e. below the detection limit (<5 pmol/L). No samples were collected before 11:00 h from participants aged 15–16.

The median times for saliva sample collection in boys and girls were 11:30 and 11:25 h, respectively. Thirty-eight per cent (462 out of 1224) and 42% (344 out of 825) of participants provided saliva samples before 11:00 h in boys and girls, respectively. Analysing within each 1-year age band, boys who had provided saliva samples before 11:00 h had higher salivary testosterone concentration (Wilcoxon–Mann–Whitney test: *P* < 0.001 for age band 12–13 years and 14–15 years; *P* = 0.015 for age band 13–14 years) compared to those who provided samples after 11:00 h (Supplementary Table 5 and Fig. 3). On the other hand, also in boys, mean salivary 11-KT concentration in the group that provided saliva samples before 11:00 h were consistently lower within every age group compared to those who supplied samples after 11:00 h. Such consistent trends for every age group in saliva collected before and after 11:00 h were not observed for A4, 17-OHP, DHEA and 11-OHA4 concentrations (Supplementary Table 5). In girls (Supplementary Table 6), there was no overall meaningful difference in mean salivary DHEA or OE2 when comparing saliva samples collected before and after 11:00 h. Overall, salivary testosterone concentrations revealed the strongest linear correlation with salivary A4 (*r* = 0.76; *P* < 0.01) while the other androgens showed a lesser degree of correlation between salivary testosterone and 17-OHP (*r* = 0.58; *P* < 0.01), 11-KT (*r* = 0.39; *P* < 0.01) or 11-OHA4 (*r* = 0.27; *P* < 0.01). Salivary 11-OHA4 concentrations showed a correlation with its precursor salivary A4 (*r* = 0.38; *P* < 0.01) and its downstream metabolite, 11-KT (*r* = 0.45; *P* < 0.01).

### Correlation between salivary steroids and PDS scores

**Figure 4:**
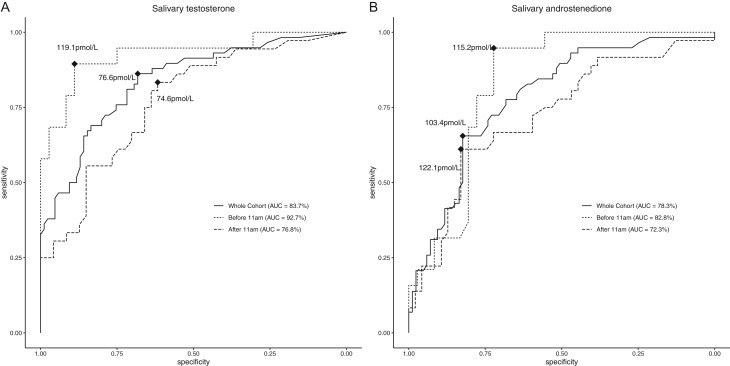
Receiver operating characteristic (ROC) curves for salivary testosterone and A4 concentrations in each specimen collection time group for predicting marked voice change in boys: (A) ROC curve for salivary testosterone concentrations collected at any time of the day, before 11:00 h and after 11:00 h. (B) ROC curve for salivary A4 concentrations collected at any time of the day, before 11:00 h and after 11:00 h. AUC is reported in brackets. Black dots and values indicate the optimal cut-off value with the boot-strapped maximum Youden index. Bootstrapped estimates for optimum cut-offs for salivary testosterone collected at any time of day were 76 and 74% for sensitivity and specificity, respectively, 84 and 89% for samples collected before 11:00 h and 75 and 66% for samples collected after 11:00 h. Bootstrapped estimates for optimum cut-offs for salivary A4 collected at any time of day were 71 and 74% for sensitivity and specificity, respectively, 83 and 72% for samples collected before 11:00 h and 61 and 79% for samples collected after 11:00 h.

Of all the salivary androgens collected from boys, testosterone had the highest correlation with the PDS composite score (*r* = 0.64, *P* < 0.01) (Table 2). In descending order, the correlations between the remaining salivary steroid concentration and PDS composite score were A4, 17-OHP, 11-KT, 11-OH-A4 and DHEA (Table 2). Salivary testosterone and A4 were also moderately correlated with advancing PDS-derived pubertal category rank (*r* = 0.63, *P* < 0.01 and *r* = 0.50, *P* < 0.01, respectively). For correlations between salivary androgens and each component in the PDS self-report: voice change was the individual component of the PDS with the highest correlation with testosterone and A4 (*r* = 0.57, *P* < 0.01 and *r* = 0.46, *P* < 0.01 respectively) (Table 2). Subgroup analyses for the time of collection revealed that correlations between testosterone and A4 with the PDS composite score, with PDS-derived pubertal categories and with almost all individual PDS components were higher for samples collected before 11:00 h (Table 2). Conversely, correlations were lower when analysis was restricted to samples collected after 11:00 h. Salivary testosterone and A4 concentrations also gradually increased with an increasing composite PDS score (Table 3). ROC curve analysis showed that specimens collected before 11:00 h had a greater discriminative performance to predict marked voice change (Fig. 4). Salivary testosterone and A4 collected before 11:00 h had an AUC of 0.93 and 0.83, respectively; when collected after 11:00 h, testosterone and A4 had an AUC of 0.77 and 0.72; when samples that were collected at all times were analysed together, the AUC was 0.84 and 0.78 (Fig. 4). By contrast, in girls, the correlation of salivary DHEA and OE2 with individual components of PDS and the PDS derived pubertal categories was much weaker (Supplementary Table 7) and the correlation between PDS composite score and salivary DHEA and OE2 was *r* = 0.21 and *r* = 0.18, respectively (*P* > 0.05).

**Table 2:** Correlations between salivary androgens and self-reported pubertal development in boys. Data are presented as Spearman correlation coefficient.

Pubertal measure	Testosterone	A4	17-OHP	11-OHA4	11-KT	DHEA
**Collected at any time**						
*n*	148	148	144	145	145	155
Individual PDS components						
Axillary hair	0.52^b^	0.44^b^	0.34^b^	0.24^b^	0.35^b^	0.11
Growth	0.32^b^	0.23^b^	0.21^a^	0.12	0.13	0.07
Facial hair	0.56^b^	0.42^b^	0.36^b^	0.1	0.21^b^	0.1
Pubic hair	0.39^b^	0.34^b^	0.22^b^	0.17^a^	0.29^b^	−0.02
Skin changes	0.18^a^	0.18^a^	0.02	0.05	0.15	0.07
Voice changes	0.57^b^	0.46^b^	0.42^b^	0.25^b^	0.36^b^	0.24^b^
Composite PDS score	0.64^b^	0.51^b^	0.39^b^	0.23^b^	0.36^b^	0.13
PDS-derived pubertal categories	0.63^b^	0.50^b^	0.45^b^	0.19^a^	0.36^b^	0.15^a^
**Before 11:00 h**						
*n*	58	58	58	56	57	62
Individual PDS components						
Axillary hair	0.53^b^	0.37^b^	0.39^b^	0.25	0.40^b^	0.08
Growth	0.37^b^	0.24	0.28^b^	−0.01	0.06	0.04
Facial hair	0.63^b^	0.40^b^	0.46^b^	0.19	0.28^a^	0.07
Pubic hair	0.53^b^	0.44^b^	0.42^b^	0.25	0.40^b^	0.02
Skin changes	0.26^a^	0.29^a^	0.14	0.06	0.09	0.03
Voice changes	0.67^b^	0.44^b^	0.50^b^	0.32^a^	0.51^b^	0.34^b^
Composite PDS score	0.75^b^	0.53^b^	0.57^b^	0.27^a^	0.45^b^	0.14
PDS-derived pubertal categories	0.69^b^	0.46^b^	0.57^b^	0.24^a^	0.42^b^	0.25^b^
**After 11:00 h**						
*n*	85	85	81	84	83	88
Individual PDS components						
Axillary hair	0.52^b^	0.46^b^	0.29^b^	0.19	0.23^a^	0.17
Growth	0.32^b^	0.22^a^	0.2	0.14	0.11	0.08
Facial hair	0.49^b^	0.42^b^	0.26^a^	0.01	0.13	0.15
Pubic hair	0.30^b^	0.30^b^	0.12	0.13	0.2	0.02
Skin changes	0.16	0.09	−0.04	−0.05	0.05	0.12
Voice changes	0.48^b^	0.41^b^	0.34^b^	0.14	0.2	0.14
Composite PDS score	0.57^b^	0.45^b^	0.29^b^	0.11	0.19	0.16
PDS-derived pubertal categories	0.58^b^	0.52^b^	0.39^b^	0.14	0.23^b^	0.12

a and b indicate statistically significant correlation coefficients where *P* < 0.05 and *P* < 0.01, respectively. Composite PDS scores were the mean of all six pubertal domain scores. The PDS-derived pubertal category in boys are derived from the sum of voice change, facial hair growth and body hair growth category from PDS self-report: prepubertal: 3, early pubertal: 4–5 (no 3-point responses), midpubertal: 6–8 (no 4-point responses), late pubertal: 9–11 and post pubertal: 12. In girls, the puberty category scores used body hair growth, breast development and menarche status as follows: prepubertal: 2 and no menarche, early pubertal: 3 and no menarche, midpubertal: >3 and no menarche, late pubertal <7 and menarche and post pubertal: 8 and menarche. Body hair growth in the present study was derived from the average scores between axillary hair and pubic hair development category in PDS self-report, rounded to the nearest integer.

11-OHA4, 11β-hydroxyandrostenedione; 17-OHP, 17-hydroxyprogesterone; A4, androstenedione; DHEA, dehydroepiandrosterone; PDS, Pubertal Development Scale.

**Table 3:** Means, standard deviation and percentiles of salivary testosterone and androstenedione (pmol/L) by composite Pubertal Development Scale (PDS) score in boys.

Composite PDS score	*n*	Mean	SD	2.5%	16%	50%	84%	97.5%
**Salivary testosterone (pmol/L)**								
1–1.49	17	13.9	17.1	3.5	3.5	6.0	29.0	41.6
1.5–1.99	27	40.8	42.6	3.5	3.9	19.0	84.5	98.6
2–2.49	45	90.0	72.9	7.1	16.2	73.0	173.4	180.6
2.5–2.99	35	126.4	74.6	18.2	70.9	119.0	188.4	194.0
3–3.49	21	182.1	133.6	13.8	48.6	158.0	328.2	355.0
**Salivary androstenedione (pmol/L)**								
1–1.49	17	59.1	35.3	24.6	37.7	51.0	71.6	79.8
1.5–1.99	27	85.6	53.4	29.6	38.3	59.0	157.1	166.6
2–2.49	45	115.9	49.1	42.2	66.1	113.0	162.0	177.6
2.5–2.99	35	148.5	60.8	49.7	88.5	138.0	226.1	231.6
3–3.49	21	153.7	97.3	29.0	78.2	134.0	243.8	332.0

Two participants had composite PDS score more than 3.5 are not displayed in the table.

### Stability analysis

A summary of stability analysis results and post-hoc estimation of degradation kinetics is available in Supplementary Table 8. Salivary testosterone, A4, 17-OHP, 11-KT and 11-OHA4 collected from the 16 male volunteers showed a significant effect of storage time (for testosterone: *P* < 0.001; A4: *P* < 0.001; 17-OHP: *P* < 0.017; 11-OHA4: *P* < 0.001; 11-KT: *P* < 0.001). Mean annual degradation as estimated from mixed effects SFO exponential decline models 6.7% for salivary testosterone (95% credible interval of (3.9, 9.4)); for A4, annual degradation 5.8% (3.0, 8.5)); for 17-OHP, annual degradation was estimated to be 7.0% (2.5, 11); for 11-OHA4, 26% per year (7.6, 42) and for 11-OHA4, 21% per year (16, 25)). Salivary DHEA, which was collected from both male and female volunteers, and salivary OE2, which was collected from females only, showed significant accumulation over the time period (*P* < 0.001 and *P* = 0.003, respectively). Post-hoc Bayesian modelling of annual accumulation of DHEA as mixed effects linear model yielded annual accumulation of +32.5% (17.5, 46.3). Mean annual accumulation of OE2 was estimated to be +1.81% (−14.8, +17.3).

## Discussion

In the current study, we have analysed the relationship between salivary sex steroids and age as well as pubertal development. To date, this is the largest study to report a wide range of pubertal sex steroid concentrations in saliva using LC-MS/MS assay in a heterogeneous group of healthy adolescents. Moreover, this study also analysed salivary sex steroid concentrations in relation to the pubertal stage using PDS self-report. We observed the discriminatory performance of salivary androgens to detect marked voice change depending on the type of androgen and the time of saliva collection. Lastly, the study examined the long-term stability of these steroids.

As expected in boys, salivary testosterone concentrations gradually increased with advancing age during pubertal years. However, compared with previous reports that measured salivary testosterone levels with LC-MS/MS method, for participants that are that are the same age, testosterone concentrations in the current study were lower (18). When compared with saliva samples collected only before 11:00 h, the difference in age-matched testosterone concentrations with previous studies was reduced emphasizing the importance of specimen collection time. Salivary androgens exhibit diurnal rhythms (5, 8, 16, 34, 35) and the magnitude of the fall in testosterone across the day depends on the age and stage of pubertal development (11, 34, 36, 37). The current study found that, in boys, salivary testosterone concentration showed a very high correlation with salivary A4. This strong correlation between the two androgens in saliva can be explained, first, by A4 being the last precursor before conversion to testosterone by 17β-hydroxysteroid dehydrogenase (17βHSD) in the testes, and second, by the expression of 17βHSD in salivary glands (38, 39). In the salivary gland, 17βHSD favours an oxidative reaction which converts testosterone to A4 locally (40). This may also account for the higher A4 concentrations compared to testosterone in saliva. Unlike salivary testosterone, salivary A4 concentration did not show a marked difference before and after 11:00 h; this difference between the two androgens has not been described before in adolescents and suggests that salivary A4 may have a greater utility when there are concerns regarding the timing of sampling.

We have demonstrated that, in the current study, the salivary testosterone and A4 concentrations in boys gradually increased in line with the composite PDS score as well as PDS-derived pubertal categories. Until now, there has been limited information on the correlation of self-reported measures of pubertal development and salivary testosterone concentration (13, 14, 41). Self-reported pubertal assessment by PDS has been recommended by several groups for pubertal assessment in population studies (23, 26, 42). The PDS can also be converted into pubertal categories which are supposed to align to Tanner-like stages (24, 25, 26, 43). Although the absolute agreement between professional Tanner Stage assessment and PDS and PDS-derived pubertal category has been reported to be low (23, 24, 25, 42), one study found that the absolute agreement between these two measurements rose from low to substantial when combining both Tanner stages and PDS-derived pubertal categories into a PDS score consisting of three broad categories; pre/early (Tanner 1–2), pubertal (Tanner 3) and late/postpubertal (Tanner 4–5) (42).

The current study shows that the composite PDS score had a high correlation with morning salivary testosterone concentration, whereas voice changes, facial, axillary and pubic hair development categories on PDS self-report had a moderate degree of correlation with salivary testosterone concentrations. These correlations with salivary testosterone were almost universally attenuated when analysis was restricted to samples collected after 11:00 h. On the other hand, salivary A4 concentrations had a moderate degree of correlation with PDS scores, irrespective of the timing of the sample. The current study identified that the voice changes category in the PDS self-report showed the strongest correlation with morning salivary testosterone concentration compared to other categorical variables of puberty development, thus highlighting the reliability of this self-reported marker of puberty in boys in estimating the timing of puberty in population studies (44). Furthermore, the ROC curves analysis showed that whenever salivary androgens (testosterone and A4) were within (lower) adult reference values (13, 14, 15), there was a high probability that the boys had marked voice change, a sign of late puberty. The discriminatory performance was greater for salivary testosterone in the morning; for salivary A4, the relationship did not change when samples were collected later in the day. Although concerns have been raised about using saliva assays to represent unbound, biologically active steroid levels due to bias from possible blood contamination (41, 45), this source of bias is reported to be rare in older children and adolescents and unlikely to significantly affect inferences (46). Taking this into consideration, our findings suggest that salivary testosterone and A4 can be reliable biomarkers for pubertal development.

A significant relationship between pubertal development using PDS self-report and salivary DHEA concentration was not detected in either sex, contrary to a previous report (23). This discrepancy between the previous report and the present study might be due to a difference in the age range of the population studied in the former, which ranged between 9 and 14 years. In addition, in girls, we found only negligible correlations between breast development, menarcheal status and salivary OE2 concentrations. This may have been due to the fact that over 75% of the girls were post-menarcheal and the timing of the sampling had not been standardized in relation to the menstrual period. Furthermore, it is possible that the OE2 assay, itself, did not reach a sufficient level of sensitivity. The 11-oxygenated C19 adrenal-derived androgens have been considered as clinically important in various human conditions including 21-OH deficiency CAH, polycystic ovarian syndrome, premature adrenarche and castration-resistant prostate cancer (47). Although 11OHA4 has minimal androgenic activity, its downstream metabolite, 11-KT has equivocal evidence on its potency to androgen receptor either lower potency (48) or equivalent potency compared to its parent steroid, testosterone (49, 50). The current study demonstrated negligible to low positive degree correlations between salivary 11OHA4, 11-KT concentration and pubertal development categories from PDS self-report in boys.

Lastly, in the stability analysis following prolonged storage at –20°C without preservatives of salivary androgens in the current study, 11-OHA4 and 11-KT showed considerable degradation, with an estimated annual degradation of 21 and 26%, respectively. Although these two steroids have previously been reported to be stable overnight at 4°C (51), we are unaware of other studies investigating long-term stability. Salivary testosterone, A4 and 17-OHP were estimated to have a maximum annual degradation of 7%. Previous studies have reported conflicting findings for the stability of salivary testosterone in long-term storage at this temperature and the discrepancy may have arisen as previous research groups have relied on immunoassays rather than tandem mass spectrometry (41, 52, 53). The former method has been shown to inflate testosterone concentration estimates when testosterone is low (less than 10 pg/mL), as is often the case in pre-pubertal boys and women (54). The extent of reduction in concentration is much smaller than the inter-individual variation of salivary testosterone and A4 collected at any one time point; therefore, despite measurable instability, we believe testosterone and A4 have utility as a marker for puberty, even when stored for long periods. Previous studies have found no measurable degradation of steroid compounds in saliva when stored at –80°C (41, 52), so it is possible that storage at –80°C may be necessary for 11-OHA4 and 11-KT, but this requires further study. For OE2 and DHEA, there was a significant effect of storage time under assumptions of F-test, which assumed for each steroid, all samples showed the same rate of concentration change. Once inter-subject heterogeneity of concentration change with storage time was considered using post-hoc linear mixed effects modelling, OE2 was estimated to have a small annual mean increase (<2%), with a large credible interval. This was most likely due to large observed heterogeneity in within-subject trajectories of OE2 concentration. The post-hoc estimation of the mean increase of salivary DHEA was over 30%. One possible explanation is possible blood contamination in saliva samples from adult volunteers used for the stability analysis; this has been reported to interfere with quantitative immunoassay-based assessment of testosterone, DHEA and OE2 (41, 45, 55). However, the likelihood of this is very low as the same observation was not observed with the salivary testosterone measurements. Furthermore, blood contamination in children and young adolescents is reported to be rare and unlikely to affect salivary hormone concentrations (46); similar low prevalences of contamination can be expected in samples collected from SCAMP participants. An alternate explanation may be due to evaporation or sublimation of water from saliva samples; this was not considered in the analysis. One method to address this would be to measure sodium (Na^+^) concentration, which has been used as a proxy for the 'evaporation constant' (56). Assuming a constant Na^+^ concentration in the samples initially, one can calculate the loss of water according to the increase in Na^+^ concentration during storage and correct analyte concentrations for samples stored over a long period.

Although the collection of samples at different times of the day posed difficulties in the interpretation of the data, subgroup analysis of the samples collected in the morning revealed that if saliva samples can be collected in the morning, testosterone is the best choice to represent puberty in boys. However, if the study is unable to collect saliva samples in the morning, then A4 may be a more attractive choice for representing puberty.

The current study did not include a correlation of the salivary samples to serum samples. However, several previous studies have demonstrated a good correlation between salivary and serum androgens (4, 57). The use of self-reported measures of puberty can be considered another limitation of the study as it may not be as accurate as Tanner staging by an experienced observer (23).

In conclusion, in boys aged 11–16 years, we have demonstrated that salivary testosterone and A4 represent valid non-invasive biomarkers that can be used as indicators for pubertal development in population studies. Furthermore, studies that rely on measuring salivary steroids on samples stored for long periods need to carefully consider the likelihood of degradation, particularly for 11-oxygenated androgens.

## Supplementary Materials

Supplementary Tables

## Declaration of interest

S Faisal Ahmed is a Senior Editor of *Endocrine Connections*. S Faisal Ahmed was not involved in the review or editorial process for this paper, on which he is listed as an author. The other authors have nothing to disclose.

## Funding

SP is funded by the Faculty of Medicine Siriraj Hospital, Mahidol Universityhttp://dx.doi.org/10.13039/501100013238, Bangkok, Thailand. AS is supported by a PhD studentship from the Medical Research Council http://dx.doi.org/10.13039/501100000265 (MRC) (grant number MR/R015732/1). The study is currently funded by the MRC (MR/V004190/1) and was originally commissioned by the Department of Health and Social Care http://dx.doi.org/10.13039/501100000276 via the independent Research Initiative on Health and Mobile Telecommunications – a partnership between public funders and the mobile phone industry (Secondary School Cohort Study of Mobile Phone Use and Neurocognitive and Behavioural Outcomes/091/0212). The SCAMP study was part supported by the MRC Centre for Environment and Health, which is currently funded by the MRC (MR/S019669/1, 2019-2024). The SCAMP study is partly funded by the National Institute for Health Research http://dx.doi.org/10.13039/100005622 (NIHR) Health Protection Research Unit in Environmental Exposures and Health and the NIHR Health Protection Research Unit in Chemical and Radiation Threats and Hazards, which are partnerships between UK Health Security Agency (UKHSA) and Imperial College London http://dx.doi.org/10.13039/501100000761 (Health Protection Research Units-2012-10141). The views expressed are those of the author(s) and not necessarily those of the MRC, NIHR, UKHSA or the Department of Health and Social Care http://dx.doi.org/10.13039/501100000276. Infrastructure support for the Department of Epidemiology and Biostatistics was provided by the NIHR Imperial Biomedical Research Centre http://dx.doi.org/10.13039/501100013342 (BRC). MBT’s Chair is supported by a donation from Marit Mohn to Imperial College London http://dx.doi.org/10.13039/501100000761 to support Population Child Health through the Mohn Centre for Children’s Health and Wellbeing. RBS’s Fellowship in Population Child Health is funded by a donation from Marit Mohn to Imperial College London http://dx.doi.org/10.13039/501100000761 to support Population Child Health through the Mohn Centre for Children’s Health and Wellbeing. The Imperial College Healthcare Tissue Bank is supported by the NIHR Biomedical Research Centre at Imperial College Healthcare NHS Trust http://dx.doi.org/10.13039/501100000762 and Imperial College London http://dx.doi.org/10.13039/501100000761.
