# Supplementary material for: Salivary androgens in adolescence and their value as a marker of puberty: results from the SCAMP cohort
Source: Endocr Connect. 2023 Nov 8;12(12):e230084. doi: 10.1530/EC-23-0084 (PMC10692692; doi:10.1530/EC-23-0084)
Supplement: Supplementary Tables [file supplementary_tables.pdf]

Supplementary tables

Supplementary Table 1. Percentile for salivary 17-OHP, 11-OHA4, 11-KT and DHEA concentrations from saliva samples collected at any time of the day in boys aged 11-16 years.

Androgens	Age	N	Mean	Percentile				
				2.5	16	50	84	97.5
17-OHP (pmol/L)	11-12	122	23.8	9.2	9.2	19.0	33.6	60.0
	12-13	473	26.6	9.2	9.2	20.0	39.0	83.2
	13-14	115	42.0	9.2	13.1	29.0	68.8	151.5
	14-15	393	40.9	9.2	17.0	34.0	64.0	111.0
	15-16	36	41.9	9.2	18.0	32.0	61.6	94.0
	11-16	1139	33.3	9.2	9.2	25.0	52.9	103.1
11-OHA4 (pmol/L)	11-12	124	206.4	50.2	85.4	163.5	283.6	454.3
	12-13	477	210.2	45.9	92.0	172.0	300.8	517.5
	13-14	118	252.9	60.0	115.1	216.5	376	650.1
	14-15	405	277.1	35.4	114	239.0	430.8	719.5
	15-16	37	230.2	35.4	61.0	200.0	347.4	778.8
	11-16	1161	238.1	35.4	97.0	195.0	352.4	649.0
11-KT (pmol/L)	11-12	121	128.4	34.0	59.0	115.0	193.6	343.0
	12-13	471	137.0	27.8	62.5	122.0	204.8	323.5
	13-14	118	197.4	40.8	96.0	179.0	279.8	461.1
	14-15	398	219.5	24.9	107.6	192.0	317.5	558.5
	15-16	37	178.1	38.6	80.8	154.0	271.7	373.7
	11-16	1145	172.3	27.6	71.0	148.0	259.0	457.2
DHEA (pmol/L)	11-12	137	784.4	12.3	37.4	294.0	844.0	4544
	12-13	518	819.7	12.3	102.0	377.5	1221.6	4587.2
	13-14	126	555.7	44.2	150.5	413.0	899.0	1996.2
	14-15	418	721.8	19.0	165.8	422.0	924.3	2652.2
	15-16	37	1139.5	49.4	160.2	676.0	2199.5	3673.9
	11-16	1236	765.3	12.3	122.0	394.0	1051.0	3290.6

Abbreviations: 17-OHP, 17-hydroxyprogesterone; 11-OHA4, 11β-hydroxyandrostenedione; 11-KT, 11-ketotestosterone; DHEA, dehydroepiandrosterone.

Supplementary Table 2. Percentile for salivary T, A4, 17-OHP, 11-OHA4, 11-KT and DHEA concentrations from saliva samples collected before 11:00 AM in boys aged 11-16 years.

Androgens	Age	N	Median	Percentile				
				2.5	16	50	84	97.5
Testosterone (pmol/L)	11-12	59	16.2	3.5	3.5	5.0	25.4	84.9
	12-13	221	40.6	3.5	3.5	19.0	89.4	174.0
	13-14	37	109.7	11.4	25.2	101.0	184.6	248.7
	14-15	109	161.9	10.5	61.2	148.0	237.2	370.0
	15-16	0	-	-	-	-	-	-
	11-16	426	74.2	3.5	3.5	35.5	158.0	282.4
Androstenedione (pmol/L)	11-12	59	56.1	18.9	25.0	46.0	71.7	171.2
	12-13	221	78.9	22.0	34.0.	61.0	115.6	192.0
	13-14	37	121.4	30.5	55.0	117.0	181.0	222.7
	14-15	109	144.9	24.0	70.2	146.0	203.7	309.4
	15-16	0	-	-	-	-	-	-
	11-16	426	96.3	21	34	80.5	163	228.5
17-OHP (pmol/L)	11-12	58	20.2	9.2	9.2	17.0	27.9	53.2
	12-13	220	28.6	9.2	9.2	23.0	43.9	93.5
	13-14	35	47.3	9.2	20.2	36.0	73.1	173.0
	14-15	106	50.4	9.2	18.0	45.5	72.0	113.0
	15-16	0	-	-	-	-	-	-
	11-16	419	34.5	9.2	9.2	26.0	54.0	111.6
11-OHA4 (pmol/L)	11-12	59	164.3	39.6	71.4	147.0	262.1	400.8
	12-13	219	208.6	40.7	81.4	155.0	297.2	510.3
	13-14	36	284.8	34.9	106.2	222.5	478.2	763.2
	14-15	108	255.7	34.2	93.0	224.0	444.6	575.6
	15-16	0	-	-	-	-	-	-
	11-16	422	221.0	33.7	82.2	165.0	333.9	607.5
11-KT (pmol/L)	11-12	59	101.1	32.0	53.5	82.0	151.6	227.6
	12-13	218	121.0	25.0	50.5	97.0.	197.0	310.1
	13-14	36	171.9	25.6	76.2	171.5	239.2	456.6
	14-15	107	183.7	21.2	74.6	162.0	262.4	404.9
	15-16	0	-	-	-	-	-	-
	11-16	420	138.6	24.5	54.0	117.0.	218.9	370.5
DHEA (pmol/L)	11-12	66	1006.9	12.3	26.0	334.0	911.8	6692.5
	12-13	245	877.6	12.3	92.2	384.0	1240.8	4530.0
	13-14	39	667.2	42.4	136.2	445.0	1069.6	2812.1
	14-15	112	870.4	26.8	158.2	410.5	924.2	2748.7
	15-16	0	-	-	-	-	-	-
	11-16	462	876.6	12.3	91.3	402	1067.7	4669.7

Abbreviations: T, testosterone; A4, androstenedione; 17-OHP, 17-hydroxyprogesterone; 11-OHA4, 11β-hydroxyandrostenedione; 11-KT, 11-ketotestosterone; DHEA, dehydroepiandrosterone.

Supplementary Table 3. Percentile for salivary T, A4, 17-OHP, 11-OHA4, 11-KT and DHEA concentrations from saliva samples collected after 11:00 AM in boys aged 11-16 years.

Androgens	Age	N	Median	Percentile				
				2.5	16	50	84	97.5
Testosterone (pmol/L)	11-12	66	15.0	3.5	3.5	6.5	32.2	66.6
	12-13	258	23.7	3.5	3.5	8.0	41.9	134.0
	13-14	81	81.6	3.5	12.0	51.0	156.2	254.0
	14-15	289	108.6	4.0	34.2	96.0	177.9	290.8
	15-16	35	135.2	7.3	43.2	128.0	200.7	338.2
	11-16	729	68.4	3.5	3.5	41.0	141.5	270.2
Androstenedione (pmol/L)	11-12	66	62.9	25.1	39.5	55.5	80.6	122.8
	12-13	258	71.0	20.9	37.0	63.0	100.9	166.7
	13-14	81	117.2	36.0	56.0	104.0	187.6	245.0
	14-15	289	139.0	28.0	79.2	132.0	190.9	291.8
	15-16	35	149.3	23.2	55.2	156.0	225.6	275.9
	11-16	729	106.1	22.0	46	90.0	169.5	257.6
17-OHP (pmol/L)	11-12	64	27.1	9.2	10.9	23.5	35.8	76.6
	12-13	253	24.9	9.2	9.2	19.0	36.0	80.4
	13-14	79	39.6	9.2	13.0	25.0.	67.6	150.0
	14-15	278	37.0	9.2	17.0	30.0	58.0	101.1
	15-16	34	42.7	9.2	17.9	32.0	62.9	99.2
	11-16	708	32.4	9.2	13.0	25.0	49.0	101.3
11-OHA4 (pmol/L)	11-12	65	244.6	72.6	96.8	182.0	300.3	883.6
	12-13	258	211.5	53.4	111.5	183.0	305.9	495.9
	13-14	81	239.6	81.0	116.0	207.0	353.4	564
	14-15	288	283.2	50.4	132.0	242.5	422.1	757.7
	15-16	35	235.8	35.4	59.5	212.0	357.4	789.2
	11-16	727	247.2	52.2	114.0	207.0	353.8	699.2
11-KT (pmol/L)	11-12	62	154.4	57.0	76.3	139.0	212.3	372.0
	12-13	253	150.8	34.0	77.2	144.0	217.8	337.0
	13-14	81	208.6	69.0	103.0	190.0	307.0	460.0
	14-15	282	233.0	38.3	119.2	200.0	335.2	564.8
	15-16	35	181.3	37.9	80.2	166.0	288.7	379.5
	11-16	713	191.7	39.6	89.0	166.0	279.1	500.2
DHEA (pmol/L)	11-12	71	577.5	12.3	70.0	279.0	707.2	3514.3
	12-13	273	767.8	12.3	103.8	364.0	1209.4	3601.6
	13-14	86	503.0	47.8	161.8	392.5	820.2	1864.8
	14-15	297	663.8	41.8	172.2	429.0	916.1	2425.6
	15-16	35	1199.8	90.6	235.1	754.0	2272.1	3795.3
	11-16	762	699.5	12.3	136.3	391.5	1023.1	3218.3

Abbreviations: T, testosterone; A4, androstenedione; 17-OHP, 17-hydroxyprogesterone; 11-OHA4, 11β-hydroxyandrostenedione; 11-KT, 11-ketotestosterone; DHEA, dehydroepiandrosterone.

Supplementary Table 4. Percentile for salivary oestradiol and DHEA concentrations from saliva samples collected at any time of the day, before and after 11:00 AM in girls aged 11-16 years.

	Age	N	Mean	Percentile				
				2.5	16	50	84	97.5
Oestradiol (All time) (pmol/L)	11-12	65	3.4	2.3	0.3	1.2	3.0	5.3
	12-13	357	3.6	2.0	0.5	1.7	3.2	5.4
	13-14	130	4.1	1.7	1.0	2.6	3.9	5.7
	14-15	292	3.9	2.0	1.0	1.9	3.7	5.6
	15-16	17	4.7	3.2	1.4	2.0	4.7	6.5
	11-16	861	3.8	2.0	0.7	1.9	3.4	5.6
Oestradiol (Before 11 AM) (pmol/L)	11-12	26	2.9	1.8	0.7	1.4	2.4	4.1
	12-13	143	4.0	2.2	0.8	1.9	3.8	6.5
	13-14	62	4.3	2.0	1.2	2.4	4.0	6.3
	14-15	113	3.8	1.6	1.2	1.9	3.6	5.2
	15-16	0	-	-	-	-	-	-
	11-16	344	3.9	2	0.8	1.9	3.7	5.9
Oestradiol (After 11 AM) (pmol/L)	11-12	39	3.6	2.5	0.3	0.9	3.4	5.7
	12-13	214	3.3	1.8	0.4	1.6	3.0	4.7
	13-14	58	4.0	1.5	1.2	2.7	3.8	5.5
	14-15	159	4.0	2.2	1.0	1.9	3.8	6.0
	15-16	17	4.7	3.2	1.4	2.0	4.7	6.5
	11-16	487	3.7	2.1	0.4	1.8	3.3	5.5
DHEA (All time) (pmol/L)	11-12	65	445.4	408.1	38	115.6	314	762.4
	12-13	357	644.5	835.4	35.4	146.4	395	1052.1
	13-14	130	516.0	608.2	71	166.0	355	761.4
	14-15	292	654.9	780.9	50.9	162.6	438	1019.4
	15-16	17	888.1	593.4	281.4	378.8	684	1528.2
	11-16	861	618.4	758.8	46.0	154.0	402	955.0
DHEA (Before 11 AM) (pmol/L)	11-12	26	397.4	377	35.9	111.0	296	716.0
	12-13	143	658.8	936.6	66.0	148.5	407	1025.5
	13-14	62	524.0	651.4	71.0	166.0	335.5	761.2
	14-15	113	747.8	948.5	48.6	147.6	465	1107.4
	15-16	0	-	-	-	-	-	-
	11-16	344	644.0	868.1	55.2	148.0	389.5	979.7
DHEA (After 11 AM) (pmol/L)	11-12	39	477.4	429.4	64.7	132.8	333.0	764.8
	12-13	214	634.9	762.5	25.9	144.2	385.5	1048.6
	13-14	58	512.2	609.7	46.2	175.7	370.0	736.8
	14-15	159	585.4	654.6	53.8	195.1	424.0	919.9
	15-16	17	888.1	593.4	281.4	378.8	684.0	1528.2
	11-16	487	600.4	685.1	24.9	161.9	395.0	937.5

Abbreviations: DHEA, dehydroepiandrosterone.

Supplementary Table 5. Median concentrations of salivary steroids collected in boys stratified by one-year age band and two-sided Wilcoxon-Mann-Whitney test.

		Collected before 11 AM		Collected after 11 AM		
	**Age Band**	N	Median Concentration pmol/L	N	Median Concentration pmol/L	*p* value
**T**	11-12	59	5	66	6	*
	12-13	221	19	258	8	**<0.001**
	13-14	37	101	81	51	**0.015**
	14-15	109	148	289	96	**<0.001**
	15-16	0	-	35	128	-
**A4**	11-12	59	46	66	56	**0.003**
	12-13	221	61	258	63	0.671
	13-14	37	117	81	104	0.507
	14-15	109	146	289	132	0.348
	15-16	0	-	35	156	-
**17-OHP**	11-12	58	17	64	24	**0.016**
	12-13	220	23	253	19	0.068
	13-14	35	36	79	25	0.084
	14-15	106	46	278	30	**<0.001**
	15-16	0	-	34	32	-
**11-KT**	11-12	59	82	62	139	**<0.001**
	12-13	218	97	253	144	**<0.001**
	13-14	36	172	81	190	0.078
	14-15	107	162	282	200	**0.001**
	15-16	0	-	35	166	-
**11-OHA4**	11-12	59	147	65	182	**0.028**
	12-13	219	155	258	183	**0.001**
	13-14	36	222	81	207	0.761
	14-15	108	224	288	242	0.092
	15-16	0	-	35	212	-
**DHEA**	11-12	66	334	71	279	0.578
	12-13	245	384	273	364	0.870
	13-14	39	445	86	392	0.633
	14-15	112	410	297	429	0.817
	15-16	0	-	35	754	-

Boldface p-values are <0.05. * For T from within 11-12 age band, this was because majority of samples were left-censored, i.e. below the detection limit (<5 pmol/L). Abbreviations: T, testosterone; A4, androstenedione; 17-OHP, 17-hydroxyprogesterone; 11-OHA4, 11β-hydroxyandrostenedione; 11-KT, 11-ketotestosterone; DHEA, dehydroepiandrosterone

Supplementary Table 6. Median concentrations of salivary steroids collected in girls stratified by one-year age band and two-sided Wilcoxon-Mann-Whitney test.

		**Collected before 11 AM**		**Collected after 11 AM**		
	**Age Band**	N	Median Concentration pmol/L	N	Median Concentration pmol/L	*p* value
**OE2**	11-12	26	2.4	39	3.4	0.239
	12-13	143	3.8	214	3.0	**0.002**
	13-14	62	4.0	58	3.8	0.735
	14-15	113	3.6	159	3.8	0.947
	15-16	0	-	17	4.7	-
**DHEA**	11-12	26	296	39	333	0.466
	12-13	143	407	214	385.5	0.466
	13-14	62	335.5	58	370	0.948
	14-15	113	465	159	424	0.480
	15-16	0	-	17	684	-

Boldface p-values are <0.05. Abbreviations: OE2, 17β-Oestradiol; DHEA, dehydroepiandrosterone

Supplementary Table 7 Correlation between salivary steroids from girls and self-reported pubertal development on PDS self-report.

Pubertal Measure	Collected at any time		Before 11 AM		After 11 AM	
	OE2	DHEA	OE2	DHEA	OE2	DHEA
**N**	63	63	33	33	29	29
**Individual PDS component**						
Axillary hair	0.32*	0.25	0.22	0.30	0.39*	0.14
Breast development	0.02	0.24	-0.21	0.24	0.27	0.26
Growth	0.00	0.08	-0.15	-0.09	0.23	0.42*
Menarche	-0.03	0.08	-0.24	-0.04	0.25	0.22
Pubic hair	0.16	0.19	0.07	0.13	0.24	0.24
Skin changes	0.12	0.07	0.12	0.06	0.04	0.08
**Composite PDS score**	0.18	0.21	0.02	0.17	0.36	0.28
**PDS-derived pubertal categories**	0.05	0.12	-0.13	0.11	0.26	0.14

Data are presented as Spearman correlation coefficient. * and ** indicate statistically significant correlation coefficients where $p<0.05$ and $p<0.01$ respectively (no p-value was below 0.01). Composite PDS score were the mean of all 6 pubertal domain scores. The PDS-derived pubertal category used body hair growth, breast development and menarche status as follows: Prepubertal: 2 and no menarche, Early pubertal; 3 and no menarche, Midpubertal: > 3 and no menarche, Late pubertal < 7 and menarche, and Post pubertal: 8 and menarche. Body hair growth in the present study was derived from the average scores between axillary hair and pubic hair development category in PDS self-report, rounded to the nearest integer.

Abbreviations; PDS, Pubertal Development Scale; OE2, Oestradiol; DHEA, dehydroepiandrosterone

Supplementary Table 8. Stability analysis results and post-hoc estimation of degradation kinetics from adult volunteers. T, A4, 17-OHP, 11-OHA4, 11-KT were collected from males only; OE2 was collected from female only; DHEA was collected from both sexes.

	Test for stability				Kinetics: estimated parameter posterior mean [95% credible interval]			
	**Df_n**	**Df_d**	**F-statistic**	**p-value**	**Model**	**Rate constant k / yr^{-1}**	**Half-life $t_{1/2}$ / yr**	**Annual change in concentration / %**
T	1	77	28.68	<0.001	SFO	0.069 [0.040, 0.098]	10.00 [7.06, 17.3]	-6.7 [-9.4, -3.9]
A4	1	77	22.19	<0.001	SFO	0.060 [0.031, 0.089]	11.60 [7.76, 22.7]	-5.8 [-8.5, -3.0,]
17-OHP	1	76	5.94	0.017	SFO	0.072 [0.025, 0.119]	9.59 [5.82, 27.7]	-7.0 [-11.0, -2.5]
11-OHA4	1	77	50.45	<0.001	SFO	0.301 [0.079, 0.547]	3.01 [2.37, 4.05]	-21.0 [-25.0, -16.0]
11-KT	1	77	8.50	0.005	SFO	0.230 [0.171, 0.293]	2.30 [1.27, 8.80]	-26.0 [-42.0, -7.6]
DHEA	1	127	51.7	<0.001	LM	-	-	+32.5 [+17.5, +46.3]
OE2	1	89	8.88	0.004	LM	-	-	+1.81 [-14.8, +17.3]

Abbreviations: T, Testosterone; A4, Androstenedione; 17-OHP, 17-hydroxyprogesterone; 11-OHA4, 11β-hydroxyandrostenedione; 11-KT, 11-ketotestosterone; DHEA, dehydroepiandrosterone; OE2, 17β-Oestradiol; Df_n, degrees of freedom of F-statistic numerator; Df_d, degrees of freedom of F-statistic denominator; SFO, single-first order kinetic model where degradation rate is assumed proportional to steroid concentration; LM, linear model where percentage analyte accumulation rate is assumed constant with respect to time.
